# Supplementary figures and images for: Development of a Pentacistronic Ebola Virus Minigenome System
Source: Viruses. 2025 May 9;17(5):688. doi: 10.3390/v17050688 (PMC12115963; doi:10.3390/v17050688)

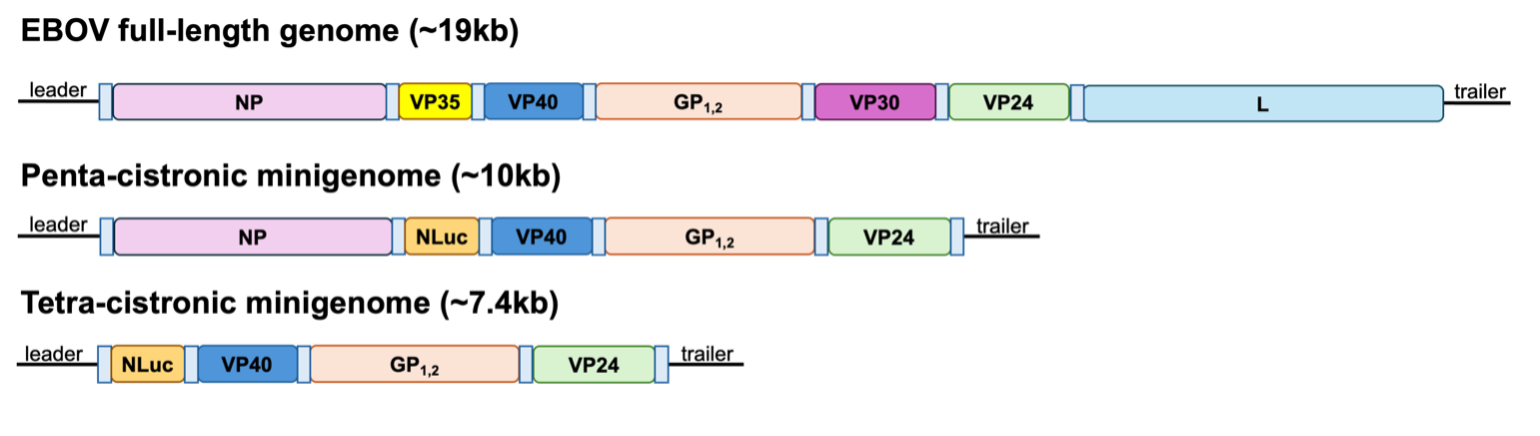

Supplement: Supplementary file 1 [file viruses-17-00688-s001.zip › Figure S1.png]

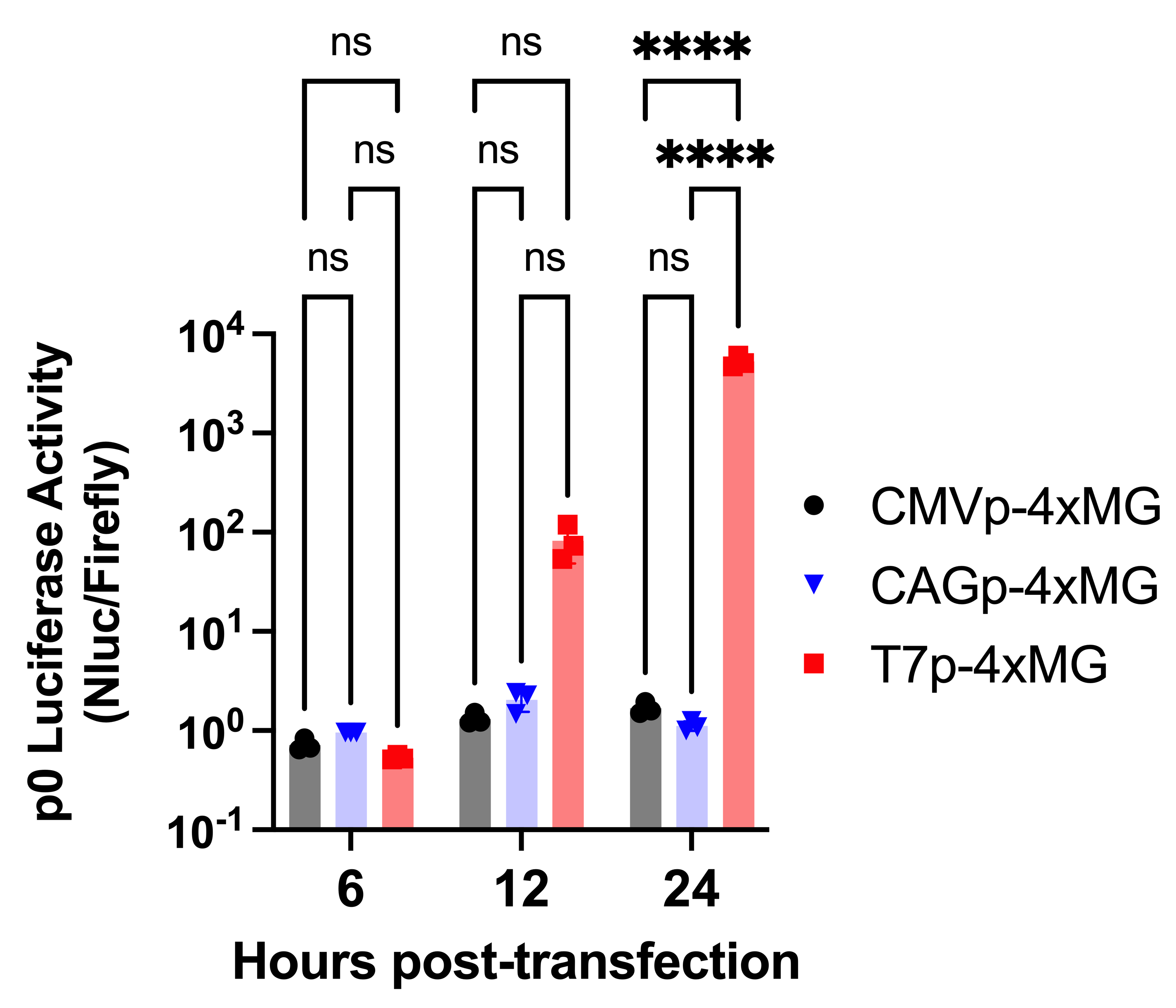

Supplement: Supplementary file 1 [file viruses-17-00688-s001.zip › Figure S2.png]

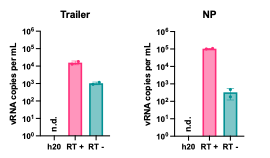

Supplement: Supplementary file 1 [file viruses-17-00688-s001.zip › Figure S3.png]
